# Regenerative Medicine: Role of Stem Cells and Innovative Biomaterials 2.0

**DOI:** 10.3390/ijms23084199

**Published:** 2022-04-11

**Authors:** Marco Tatullo, Adriano Piattelli, Barbara Zavan

**Affiliations:** 1 Department of Basic Medical Sciences, Neurosciences and Sense Organs, University of Bari Aldo Moro, 70124 Bari, Italy; 2 Honorary Senior Clinical Lecturer, University of Dundee, Dundee DD1 4HR, UK; 3 Founding Member of MIRROR—Medical Institute for Regeneration and Repairing and Organ Replacement, Interdepartmental Center, University of Bari Aldo Moro, 70124 Bari, Italy; 4 School of Dentistry, Saint Camillus International University of Health and Medical Sciences, Via di Sant’Alessandro 8, 00131 Rome, Italy; adriano.piattelli@unich.it; 5 Department Translational Medicine, University of Ferrara, 44121 Ferrara, Italy; barbara.zavan@unife.it

Regenerative medicine has constantly increased its field of influence over the last few years. Basically, materials of tissue regeneration are stem cells and novel and smart biomaterials. Nevertheless, the study models involving stem cells and regenerative procedures are even more complex, and the results achieved are promising. 

Mesenchymal stem cells (MSCs) have been shown to improve liver function in both acute and chronic liver disease models; an interesting study has evaluated the role of allogenic MSC transplantation in a porcine model subjected to repeated biliary obstruction followed by partial hepatectomy, demonstrating that MSC transplantation promoted the growth of the damaged liver tissue [1]. 

Due to their anti-inflammatory, immunomodulatory, and regenerative capacity, mesenchymal stem cells are considered to be an interesting tool in several biological models. Of course, the multifaceted activity of MSCs may suggest also using them in some specific critical conditions, such as in the regeneration of ex situ normothermic machine perfused organs, to expand the donor pool and improve the quality of organs to be transplanted [2].

To date, worldwide researchers have been investigating and comparing the main protocols able to ensure safe and predictable stem cell-based procedures; among the most promising tissue models, bone tissue is one of the most studied [3]. Bone tissue engineering combined with stem cell applications represents a reliable opportunity to improve the medical fields where bone tissue is necessary to ensure function and aesthetics [4]. In this landscape, the osteogenic potential of stem cells and induced pluripotent stem cells (iPSCs) has been found to be promising in dental [5] and orthopedic [6] diseases. Importantly, the vast majority of MSC-rich seats are difficult to reach, except during surgical treatments; for this reason, MSCs are easily achievable, and according to particularly accessible sources, they are even more appreciated in modern protocols [3]. On the other hand, not all MSCs are capable of differentiating between site-specific tissues; adipose stem cells (ASCs) have shown efficient chondrogenic differentiation, given physical and mechanical stimuli [6], thus making such cells promising in clinical applications. Nevertheless, because these cells are considered “drugs” in Europe and the USA, such restrictions have pushed new protocols for obtaining and using minimally manipulated ASCs. Undoubtedly, the trends that work “without” specific factors, such as cells (cell-free protocols) or scaffolds (scaffold-free approaches), are intriguing, but often poorly reliable in clinical applications; in fact, bone defects are typically critical-size defects (CSDs): this fact makes it quite difficult to use scaffold-free approaches. On the other hand, several strategies have been developed to improve traditional biomaterials and to discover novel materials to be applied to medical fields. Due to its mechanical behavior and safety in bone tissue repair, titanium (Ti) is widely used both in orthopedics and dentistry. Recently, it has been reported that the functionalization of Ti surfaces with the tetrapeptide KRSR (Lys-Arg-Ser-Arg) is able to improve osteoblasts’ adhesion to prosthetic surfaces [7]. Currently, the biological regulation induced by traditional materials decorated with bioactive molecules is carefully compared with the naïve properties shown by novel promising biomaterials, such as graphene and the recently discovered phosphorene and borophene [8,9].

In conclusion, there is a strong consensus on the need for more user-friendly regenerative procedures. The role of novel cells, novel biomaterials, and their interaction with the microenvironment will be even more important in future regenerative and reparative processes. Growing evidence is showing how, in the future, we are going to regenerate human tissues with little/no cell manipulation. This Special Issue has certainly reported a clear overview of the most promising future regenerative applications: the forthcoming years will be critical when it comes to translating such concepts from bench to bedside. 

## References

[1] Vištejnová L., Liška V., Kumar A., Křečková J., Vyčítal O., Brůha J., Beneš J., Kolinko Y., Blassová T., Tonar Z. (2021). Mesenchymal Stromal Cell Therapy in Novel Porcine Model of Diffuse Liver Damage Induced by Repeated Biliary Obstruction. Int. J. Mol. Sci..

[2] Bogensperger C., Hofmann J., Messner F., Resch T., Meszaros A., Cardini B., Weissenbacher A., Oberhuber R., Troppmair J., Öfner D. (2021). Ex Vivo Mesenchymal Stem Cell Therapy to Regenerate Machine Perfused Organs. Int. J. Mol. Sci..

[3] Tatullo M., Codispoti B., Paduano F., Nuzzolese M., Makeeva I. (2019). Strategic Tools in Regenerative and Translational Dentistry. Int. J. Mol. Sci..

[4] Tatullo M. (2018). About stem cell research in dentistry: Many doubts and too many pitfalls still affect the regenerative dentistry. Int. J. Med. Sci..

[5] Hollý D., Klein M., Mazreku M., Zamborský R., Polák Š., Danišovič Ľ., Csöbönyeiová M. (2021). Stem Cells and Their Derivatives—Implications for Alveolar Bone Regeneration: A Comprehensive Review. Int. J. Mol. Sci..

[6] De Francesco F., Gravina P., Busato A., Farinelli L., Soranzo C., Vidal L., Zingaretti N., Zavan B., Sbarbati A., Riccio M. (2021). Stem Cells in Autologous Microfragmented Adipose Tissue: Current Perspectives in Osteoarthritis Disease. Int. J. Mol. Sci..

[7] Tarjányi T., Bogár F., Minarovits J., Gajdács M., Tóth Z. (2021). Interaction of KRSR Peptide with Titanium Dioxide Anatase (100) Surface: A Molecular Dynamics Simulation Study. Int. J. Mol. Sci..

[8] Tatullo M., Genovese F., Aiello E., Amantea M., Makeeva I., Zavan B., Rengo S., Fortunato L. (2019). Phosphorene Is the New Graphene in Biomedical Applications. Materials.

[9] Tatullo M., Zavan B., Genovese F., Codispoti B., Makeeva I., Rengo S., Fortunato L., Spagnuolo G. (2019). Borophene Is a Promising 2D Allotropic Material for Biomedical Devices. Appl. Sci..

